# Psychosocial Consequences of Sexual Assault on Women: A Scoping Review

**DOI:** 10.1007/s10508-024-03013-1

**Published:** 2024-10-30

**Authors:** Ester Serrano-Rodríguez, Violeta Luque-Ribelles, Vanesa Hervías-Parejo

**Affiliations:** 1 Social, Criminological and Behavioral Science, University of Cadiz, Cádiz, Spain; 2 Department of Psychology, Facultad de Ciencias de la Educación, University of Cadiz, Campus de Puerto Real, Av. República Saharaui s/n, 11519 Puerto Real, Cádiz, Spain; 3 INDESS, Instituto Universitario de Investigación para el Desarrollo Social Sostenible, Jerez de la Frontera, Spain; 4 Department of Labour Law and Social Security, University of Cadiz, Jerez de la Frontera, Spain

**Keywords:** Post-traumatic stress disorder, Psychosocial consequences, Scoping review, Sexual assault, Women

## Abstract

Women are at a higher risk of sexual assault, partly due to societal sexism. This scoping review addresses the question: What is known about the psychosocial consequences of sexual assault on women? To understand these impacts, various studies were reviewed. Searches were conducted in Web of Science, PubMed, Scopus, PsycArticles, and PsycINFO between April and May 2022. The methodology followed Arksey and O’Malley’s (2005) framework. PRISMA guidelines were used for screening and the Scoping Reviews Checklist was employed to ensure the quality of the review process. As a result, 1744 studies were identified, and 21 were selected based on inclusion and exclusion criteria, with publication years ranging from 1997 to 2021. The total sample of women analyzed across these studies was 20,071 (*M* = 955.76; *σ* = 2236.53). These 21 studies examined the psychosocial consequences of sexual assault on women's lives. The primary psychosocial outcomes identified were depressive symptoms (*n* = 13), anxiety symptoms (*n* = 11), post-traumatic stress disorder (*n* = 11), alcohol dependence (*n* = 6), and sleep disorders (*n* = 3). In conclusion, this review enhances our understanding of the effects of sexual assault on women and identifies areas for future research to further understand and address this phenomenon. This work can ultimately guide efforts to improve the well-being of survivors.

**Supplementary Information:**

The online version contains supplementary material available at 10.1007/s10508-024-03013-1.

## Introduction

Being a woman significantly increases the likelihood of being a victim of sexual assault (Cruz, 2017). The World Health Organization (WHO) and Pan American Health Organization (PAHO) define sexual violence as: “Any sexual act, attempt to obtain a sexual act, unwanted sexual comments or advances, or acts to traffic or otherwise directed against a person’s sexuality using coercion, by any person regardless of their relationship to the victim, in any setting, including but not limited to home and work” (WHO & PAHO, 2012, p. 2). It is estimated that 15% of women will experience sexual assault in their lifetime (Campbell & Wasco, 2005; Echeburúa et al., 2013; Pinsky et al., 2017; Vickerman & Margolin, 2009). Globally, approximately 6% of women (aged 15–49 years and older) have been victims of sexual assault by someone other than a partner at least once in their lifetime. Additionally, nearly 30% of women in relationships worldwide have experienced physical and/or sexual violence by an intimate partner (WHO, 2021). Sexual assault is an umbrella term that includes various forms of sexual violence, including rape (Young & Maguire, 2003). The term "rape" encompasses vaginal, oral, and anal penetration (Luce et al., 2010), meaning all types of non-consensual penetration (Pinsky et al., 2017).

Experiencing sexual assault likely has severe negative consequences on a woman's life, including adverse physical and psychological health outcomes, difficulties in daily activities, and an increased risk of repeated sexual assault (Kilpatrick et al., 2007; Koss et al., 2003). Specifically, the psychological impact of sexual assault includes significant issues like post-traumatic stress disorder (PTSD) and depressive symptoms (Ullman et al., 2007), which can negatively affect women's sexual health and functioning (Rellini, 2008; Van Berlo & Ensink, 2000). Survivors may experience immediate psychological effects such as anxiety, fear, emotional instability, disbelief, guilt (Luce et al., 2010), and suicidal risk (Newins et al., 2021). Studies have also shown that women veterans who have experienced military sexual assault are at a higher risk of suicidal ideation due to PTSD than those who have PTSD from other types of trauma (Blais & Monteith, 2019). Additionally, 25% of people who have been victims of violent behavior develop PTSD, but this percentage rises to 50–60% for women who have been sexually assaulted (Echeburúa et al., 1997). PTSD occurs when a person experiences or witnesses a physical assault or threat to their own or another's life, leading to an intense reaction of fear, horror, or helplessness (DSM-5). Symptoms of PTSD include negative changes in cognition and mood, re-experiencing, and avoidance (American Psychiatric Association, 2013). Trauma leaves a lasting imprint on the mind, brain, and body, affecting how individuals cope in the present (van der Kolk, 2014). Sexual violence against women is a global issue with severe consequences for survivors. Beyond psychological impacts, women who are victims of sexual violence also face social consequences. Psychosocial consequences are understood as the interplay between psychological and social factors on mental health and behavior (Vizzotto et al., 2013). Psychological factors include internal resources such as self-esteem, personal autonomy, impulse control, empathy, and a sense of humor. Social factors involve contextual influences like interpersonal relationships, demographic characteristics, and social structures, including culture and environment (Grotberg, 1995). Addressing both psychological and social consequences is crucial to mitigating the harmful effects of sexual violence.

### Study Aims

This scoping review aims to analyze the scientific literature on the psychosocial impact of sexual assault on women. Specifically, the research focuses on answering the question: What is known about the psychosocial consequences of sexual assault on adult women? Understanding the impact of this traumatic event will provide an overview of the main consequences on women's lives.

## Method

The methodology for this scoping review followed Arksey and O'Malley's (2005) framework, consisting of five stages: identifying the research question, identifying relevant studies, study selection, charting the data, and collating, summarizing, and reporting the results. Additionally, the PRISMA Statement (Page et al., 2021) and the Preferred Reporting Items for Systematic Reviews and Meta-Analyses extension for Scoping Reviews (PRISMA-ScR) Checklist (Tricco et al., 2018) (https://www.prisma-statement.org/) were utilized (see Supplementary material).

### Search Strategy

Comprehensive literature searches were conducted by two researchers using the following search string: ("sexual assault" OR "rape") AND "women" AND ("psychosocial impact" OR "impact" OR "consequences"). Searches were limited to peer-reviewed academic journals in English and Spanish and were conducted between April and May 2022 in Web of Science (WOS), PubMed, Scopus, PsycArticles, and PsycINFO. No time period restrictions were applied.

To test the suitability of the research question, the term "adult sexual assault" was included in PROSPERO (International Prospective Register of Systematic Reviews), yielding four articles, only two of which analyzed the psychosocial impact of sexual assault on adults (male and female, and female-serving military and veteran populations) (Campbell et al., 2022; Rollison et al., 2021). The results indicate that recent research has been conducted in this area, and this review aims to contribute further to this knowledge.

### Eligibility Criteria

The inclusion criteria for the study were as follows: (1) the population consisted of women victims of ASA; (2) the outcome variables included psychological and/or psychosocial consequences on women's health; (3) the articles referred to sexual assault, rape, or sexual harassment.^1^ Exclusion criteria included: (1) articles not written in English or Spanish; (2) articles analyzing child sexual assault (CSA); (3) articles that did not differentiate between men and women who had been sexually assaulted; (4) systematic reviews, literature reviews, annual reviews, clinical reviews, books, dissertations, commentaries, and meeting/conference abstracts and proceedings.

### Charting Data

The analyses process began by extracting the titles and abstracts of all articles identified through the database searches. This information was compiled into a Microsoft Excel document. Subsequently, the selected papers were analyzed based on: author(s) and study ID, design, settings, participants, measures, and main results, and this information was summarized in a Microsoft Word table. The analyses involved one researcher screening the databases and articles, followed by a double-independent analyses process by two investigators. The articles were divided into two groups, and each investigator analyzed their assigned group before exchanging them. Any discrepancies were resolved through consensus. For studies with multiple objectives, only information relevant to the review's objective was analyzed (e.g., Study 9 and Study 21). An external third researcher provided an assessment of the methodological and analytical process and contributed to the content of the article.

## Results

**Fig. 1:**
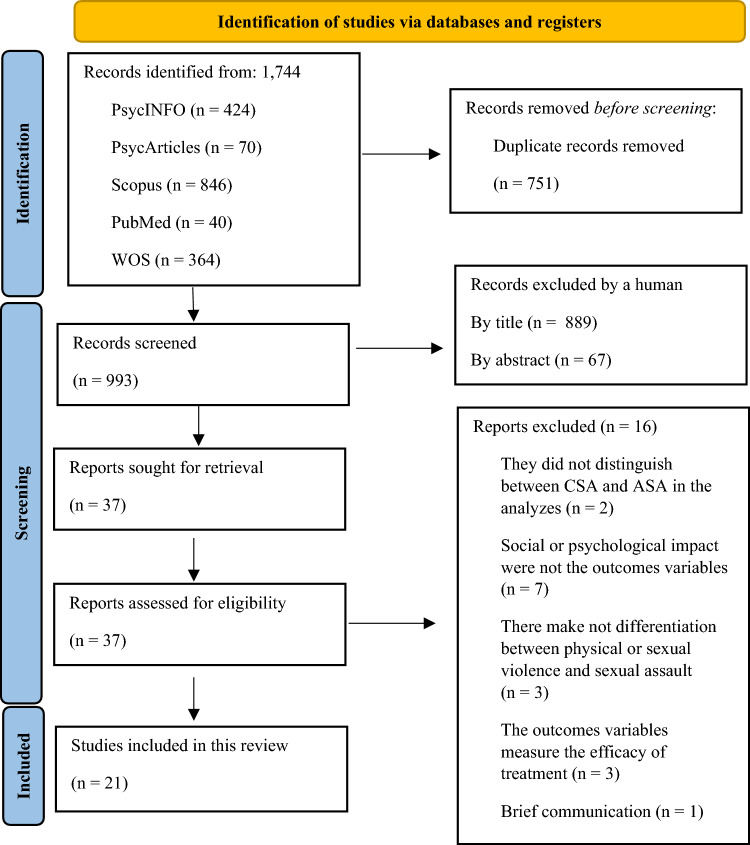
PRISMA diagram. Source: Adapted from Page et al. (2021)

The search returned a total of 1744 studies on sexual assault, of which 751 were discarded as duplicates. Of the remaining 993 studies, 889 were excluded based on titles indicating they were outside the review's scope. A further 67 papers were discarded after abstract review due to irrelevance to psychological and/or psychosocial consequences. This left 37 studies, of which 16 were excluded for various reasons: CSA analyses (*n* = 2), absence of psychosocial outcomes (*n* = 7), no distinction between types of violence (*n* = 3), drug treatment effectiveness outcomes (*n* = 3), and brief communication (*n* = 1). Ultimately, 21 studies were included in the review, meeting all inclusion and exclusion criteria, and encompassing a total sample of 20,071 women. The smallest sample size was 25 women, and the largest was 10,171 (*M* = 955.76; *σ* = 2287.93). The PRISMA flow diagram illustrates the screening and selection process (Fig. 1).

The studies are numbered from Study 1 to Study 21 for reference. Table 1 provides detailed information on the studies, including design, scope, participants, measures, and main results. Eleven studies were conducted in the USA (Studies 2, 3, 8, 9, 11, 12, 13, 14, 17, 18, and 19), two in the UK (Studies 15 and 20), one in South Africa (Study 1), one in Colombia (Study 4), one in Spain (Study 5), one in Italy (Study 6), one in Turkey (Study 7), one in Croatia, Bosnia, and Herzegovina (Study 10), one in India (Study 16), and one in Brazil (Study 21). The studies utilized the following designs: cross-sectional (*n* = 12), with 7 explicitly stated (Studies 2, 6, 7, 8, 9, 16, and 19) and 5 inferred (Studies 1, 11, 14, 15, and 20). Longitudinal designs were present in 7 studies, with 2 explicitly stated (Studies 13 and 17), and 1 specifying longitudinal prospective design (Study 3). Study 10 had a longitudinal design, Study 18 applied a longitudinal repeated measurement design, and Study 21 reported a longitudinal design with a control group. Study 4 utilized a retrospective and prospective design, while Study 12 reported both cross-sectional and longitudinal designs. The duration of the longitudinal designs varied from four weeks (Study 1) to 3 years (Studies 9 and 14). All studies employed quantitative methods, with sample sizes ranging from 25 (Study 20) to 10,171 (Study 4). Participant ages ranged from 15 years (Study 15) to 83 years (Study 10). The studies used socio-demographic data such as race, ethnicity, and nationality interchangeably.

**Table 1:** Characteristics of included studies

Study	Study design	Scopes	Participants	Measures	Main results
Study 1. Abrahams et al. (2013)	Cross-sectional (n.s.)	To explore depression symptoms four to six weeks after rape and whether these differ according to the circumstances of the rape.	The participants (*n* = 140) were HIV negative adults and adolescents (mean age: 21). The majority had 10 or more years of education. 1/3 was employed. Recruited: from public hospital services in the Eastern and Western Cape (South Africa) from an intervention study to improve adherence to post-exposure prophylaxis for HIV after a sexual assault. Eligibility criteria: negative HIV test, acceptance of the PEP medication and having their own cell phone. Exclusion criteria: not seek consent from participants who were emotional distressed at the time of the recruitment at the rape centers.	Structured questionnaire (sociodemographic characteristics, information related to sexual assault, PEP medication, assistance, and support received and experiences of side effects of the drugs). Variables on rape: relationship to the perpetrator, number of perpetrators, use of a weapon during attack and contact with the perpetrator after the rape. Center for Epidemiological Studies Depression Scale (CES-D scale).	Response rate of 92.3%. 27.9% were from the Eastern Cape. 59.3% knew the perpetrator. In 1/6 of the perpetrators were an intimate partner (16.7%). 42.9% were raped by multiple perpetrators. 84.3% (95% CI: 78.1–90.3) had a depression symptom score of 16 or over. Women whose perpetrator was a stranger had a lower likelihood of depression symptoms. Those experiencing more than 4 side effects had a greater likelihood of very high levels of depression symptomatology (score < 22). There was a greater likelihood of reporting very high levels of depression symptoms among Colored and African women from Cape Town compared to African women from the Mthatha site for the higher score. Further analyses by race showed significant differences in rape-related factors between sites and racial groups. Most of the rape with weapons and multiple perpetrator rape was experienced by African women.
Study 2. Bedard-Gilligan et al. (2011)	Cross-sectional	To examine the relationships between sexual assault, subsequent drinking behavior and consequences, and alcohol expectancies.	306 undergraduate women enrolled at a large west-coast university from US, who reported current alcohol use and reported either no trauma history (*n* = 53), non-AIA (alcohol-involved assault) (*n* = 69), or AIA (*n* = 184). Inclusion criteria: endorsing at least some heavy episodic drinking. Exclusion criteria: women who reported both an AIA and a non-AIA (*n* = 84). Participants mean age was 20.38 years (SD = 1.50). Most participants were Caucasian (72.5%). 16.0% being Asian/Pacific Islander, 2.0% being African American, 1.0% being Native American/Alaskan Native, 7.5% being multi-ethnic, and 3% reporting other as their racial background. 4% of participants reported Hispanic ethnicity. 96.4% participants identified as heterosexual, with 1.3% identifying as bisexual, 0.3% identifying as lesbian, and 2.0% identifying as questioning.	Self-report measures of demographics (sexual assault experiences, alcohol use, alcohol expectancies and consequences of alcohol use). Standardized Trauma Interview (STI). The Quantity Frequency Questionnaire (QF). Information to compute blood alcohol content (BAC). Modified version of the Daily Drinking Questionnaire (DDQ). Comprehensive Effects of Alcohol Questionnaire (CEOA). Modified version of the Rutgers Alcohol Problem Index (RAPI).	Differences emerged for alcohol use (*F*(2, 298) = 12.78, *p* < .001), peak blood alcohol content (*F*(2, 298) = 9.66, *p* < .001), consequences (*F*(2, 296) = 7.38, *p* < .005), and positive alcohol expectancies (*F*(14, 796) = 1.93, *p* < .05). Women with an AIA reported greater alcohol use and positive expectancies compared to women with no trauma history and women with a non-alcohol influenced assault. Both assault groups reported greater drinking consequences than women with no trauma history.
Study 3. Carey et al. (2018)	Prospective longitudinal	To examine the effect of sexual assault in the first semester on mental health at the end of that semester, controlling for baseline sexual assault and mental health.	483 first-year female college students at a private university in the north-eastern U.S. Women > age 25 or < age 18. 94% of women were 18 years old, 29% were in a committed relationship and 96% reported their sexual orientation as heterosexual. 64% identified as white, 11% as Asian, 10% as Black, 9% as Hispanic, and 13% as other or multiple races.	Questionary about age and race/ethnicity at baseline. Sexual Experiences Survey-Revised (SES-R); Patient Health Questionnaire-9 (PHQ-9); Generalized Anxiety Disorder-7 (GAD-7).	Women who experienced first-semester sexual assault presented clinically significant levels of depression (χ^2^(1, *N* = 419) = 5.41, *p* = .020), and anxiety symptoms at the end of the first semester (χ^2^(1, *N* = 419) = 3.94, *p* = .047).
Study 4. Domenech del Río and Sirvent (2017)	2-fold: (1) Retrospective review of survey data. (2) Prospective interview data.	To analyze the mental health consequences of non-partner sexual violence.	A nationally representative sample of 10.171 women from a Spanish survey carried on in Spain. Three mutually exclusive categories of non-partner sexual violence were created to measure the effects of violence on health ([1] who reported sexual touching or other forms of non-partner sexual violence; but not a rape or a rape attempt; [2] those who disclosed a rape attempt; [3] women who reported a rape with or without a rape attempt or sexual touching or other forms of no partner sexual violence).	Spanish Survey on Violence against Women (2015) (although it is not an instrument, the data are collected from this national survey).	All the categories of non-partner sexual violence were strongly associated with the different health outcomes. Rape increased the likelihood of reporting anxiety [odds ratio, OR: 3.77 (2.65–5.37)], sadness because of feelings of worthlessness [OR: 3.31 (2.32–4.73)]; and the desire to cry without reason [OR: 3.46 (2.45–4.89)] more than 3-fold. The relationship of the victim with the perpetrator varied by the type of sexual victimization.
Study 5. Escobar-Córdoba and Eslava-Schmalbach (2012)	Non-experimental, prospective matched double cohort study. (Longitudinal, n.s.)	To determine the presence of sleep disturbance and PTSD symptoms in rape victims and to assess the causal influence of exposed and unexposed groups and compare the respective events.	68 women living in Bogotá who went to a Forensic Medicine Institute. Participants were 34 women raped victims (women > age 45 or < age 18), and 34 women not exposed (aged ranged +/− 5 years within the exposed victim).	Pittsburgh Sleep Quality Index (PSQI); Epworth Sleepiness Scale (ESS); Impact of Event Scale (IES).	They found significant differences in the total score of sleep quality variables and PTSD symptoms. This variables in women victims of raped are significant in comparison with women non-raped group. Sleepiness 35.29%; sleep disturbances, 52.94%; trouble sleeping due to: having to get up to go to the bathroom 26.47%, unable to breathe well 29.41%, coughing or snoring loudly 26.47%; and daytime dysfunction, 55.88% that potentially can be correlated with SDB, and PLMD.
Study 6. Faravelli et al. (2004)	Cross-sectional with control group (n.s.)	To study the psychopathological consequences of a single rape in adult female victims.	40 adult women victims of rape (mean age: 34, 5 years the time of the rape). 85% were raped by a single man, and 15% were raped by 2 or more men. The aggressors were known to the victims in 4 cases. At the time of the rape, mean age was 34.5 years (SD = 7.4). 55% were married, 20% were separated or divorced, and 25% were unmarried. 32 women were experimented a life-threating trauma but nonsexual events (control group) (mean age: 33, 7 years). 53% were married, 22% were separated or divorced, and 25% were unmarried. 13% had experienced a car accident with risk for the life, 56% had experienced a physical assault, and 31% were violently robbed during the previous 9 months. None of them underwent sexual abuse during childhood or adolescence. Recruited: raped women were recruited from a women's association dedicated to the assistance of battered women. Control group: 32 women from the general population who had suffered a life-threatening trauma (except any form of sexual abuse). Participants were recruited by records of a community study conducted in Sesto Fiorentino.	Florence psychiatric interview	Women victims of rape showed a significantly greater prevalence of PTSD (*N* = 38, 95%, vs. *N* = 15, 47%) (χ^2^ = 21.2, df = 1, *p* = 0.000005), and sexual disorder (*N* = 36, 90%, vs. *N* = 6, 19%) (χ^2^ = 33.2, *df* = 1, *p* = 0.000001), eating disorder (*N* = 21, 53%, vs. *N* = 2, 6%) (χ^2^ = 16.2, *df* = 1, *p* < 0.00006), major depression (*N* = 30, 75%, vs. *N* = 14, 44%) (χ^2^ = 5.8, *df* = 1, *p* < 0.02). Anxiety disorders (excluding PTSD) were also more prevalent in the raped group, although they were at the limit of significance (*N* = 15, 38%, vs. *N* = 5, 16%) (χ^2^ = 3.7, df = 1, *p* = 0.054).
Study 7. Karataş et al. (2020)	Cross-sectional cohort study	To investigate pre-, peri- and PTSD factors associated with post-PTSD in adult women who had been sexually assaulted.	60 adult women who had been sexually assaulted (mean age:27.35 years, SD ± 6.43). Mean duration of education was estimated to be 10.35 years (SD ± 3.12), and monthly income was estimated to be 1162.84 (SD ± 793.62) Turkish Liras. Just over 42% of the participants were single and primary or secondary school graduates, and most were unemployed (70%). 52% of the participants had no history of mental disorder, and about 2/3 no history of self-harm (65%), or suicide attempts (68%). Recruited: all women attending a specialist university clinic for victims of sexual assault referred by the courts for a mental health assessment in Turkey.	Semi-structured interview to collect sociodemographic information. Multidimensional Scale of Perceived Social Support (MSPSS); Traumatic Stress Symptom Checklist (TSSC); Beck Depression Inventory (BDI); Beck Anxiety Inventory (BAI); Medical Outcomes Study Short Form-36 (SF-36).	68% were found to have PTSD, between them was significantly more likely rated themselves as of lower socioeconomic status. There was no association between conditions at the time of the assault or assailant characteristics, and development of PTSD. About to post-assault characteristics, there was a trend toward the group presenting with PTSD to have been more likely to have had suicidal thoughts. They were significantly more likely to have scale scores indicative of depression, anxiety, and to record other health problems measurement by the Short Form-36 (SF-36) (e.g., sense of poor general health, impaired social and physical function, impaired emotional role). Women with PTSD had rated themselves as feeling a level of social support that was significantly lower than that recorded by the women without PTSD. Differences between the women with and without PTSD were significant only in the forced migration and economic difficulties categories. Correlational analyses confirmed strong relationships between PTSD, and other anxiety and depressive symptoms, and more modest but significant relationships with the other scaled measures of concurrent disorders, or functional levels. Perceived social support and presence of suicidal thoughts following assault were independently related to the experience of PTSD.
Study 8. Lindquist et al. (2013)	Cross-sectional	To examine the context of sexual assault, and post-assault actions, and consequences among women attending Historically Black Colleges or Universities (HBCUs).	A web-based surveys of 3951 undergraduate women at four HBCUs (US). 51, 6% between 18 and 20 years. 87.2% of the women were Black. Participants were women who: had been sexually assaulted since entering college (*n* = 358); had experienced completed physically forced sexual assault since entering college (*n* = 188); and who had experienced incapacitated sexual assault since entering college (*n* = 250).	HBCU-CSA study; Center for Epidemiologic Studies—Depression (CES-D 10) scale; slightly modified version of the of the Primary Care PTSD (PC-PTSD) screen.	Survivors were more likely to screen positive for PTSD (53% of survivors of incapacitated sexual assault, and 50% of survivors of physically forced sexual assault) than nonvictims (23% of women), and had mean depressive symptom scores of 16 (compared to 12.5 among women who had not been assaulted). For both types of assault, who reported sustaining an injury during the assault had significantly higher odds of screening positive for PTSD than who did not sustain an injury (for incapacitated sexual assault survivors, OR = 2.5, 95% CI [1.3, 4.7]; for physically forced sexual assault survivors, OR = 2.0, 95% CI [1.03, 3.9]). Who had been victim of incapacitated sexual assault and reported an injury also had significantly more symptoms of depression than those who did not (*b* = 3.37, *p* < .01).
Study 9. Littleton et al. (2013)	Cross-sectional	To investigate sexual assault, and health risk behaviors (hazardous drinking, and engaging in sexual behaviors to regulate negative affect) with a large, ethnically diverse sample of college women.	Participants were drawn from a sample of 1744 women enrolled at one of the three U.S. southeastern universities who completed an online survey for course credit across two academic semesters. The sample was restricted to the 1620 women (92.9% of the sample).72.3% described themselves as European American, 8.8% as African American, 8.9% as Latina, and 10% as Asian American. 20% of participants had been a victim of completed sexual assault. (mean age: 20.5 years). Among participants, 3.9% European American women, 9.8% African American women, 29.2% Latina women, and 35.2% Asian American women reported that they were U.S. immigrants.	Ethnicity was determined by self-report. Sexual Experiences Survey (SES), 2 items; Binge drinking prior to the assault assessed with 2 items; Center for Epidemiological Studies Depression Scale (CES-D); Four-Dimensional Anxiety scale (FDAS); Alcohol Use Disorders Identification Test (AUDIT); Use sex to Reduce Negative Affect scale.	Depression and anxiety were examined as mediators of the relationship between sexual assault and health risk behaviors. Between European American women were evidence of moderated mediation. Among all ethnic groups, the relationship between sexual assault, depression and anxiety was mediated by the use of sexual behavior as an affect regulation strategy.
Study 10. Lôncar et al. (2006)	Retrospective longitudinal	To explore the short- and long-term psychological consequences of rape on women victims during war against Croatia, and Bosnia and Herzegovina.	68 women victims of rape aged between 14 and 83 years (mean age: 32 +/− 6.4 years). The women entered the study on average (+/−standard deviation) 11.9 +/− 2.4 months after being raped or released from captivity during which they had been raped. 44 of them were raped more than once, 21 were raped every day during their captivity, and 18 were forced to witness rapes. Recruited by: Center workers offering professional help to the war victims and individual contacts at Croatian refugee camps in the eastern Slavonia, and Zagreb.	Testimony method. Questionnaire of 44 multi-choice questions about socio-demographic data, type and nature of the sexual torture, and acute stress reaction. Structured clinical interview: according to the DSM-III classification.	Most common psychological symptoms suffered immediately after the rape were depressiveness (*n* = 58), avoidance of thoughts or conversations associated with the trauma (*n* = 40), suicidal ideas (*n* = 25), depression (*n* = 52), social phobia (*n* = 51), PTSD (*n* = 21), and sexual dysfunctions (*n* = 17). Out of 29 women who got pregnant after rape, 17 had artificial abortion. This decision was strongly predicted by suicidal thoughts, and impulses.
Study 11. McConnell et al. (2020)	Cross-sectional (n.s.)	To investigate the incidence of combined and incapacitated rape among adult female rape survivors, as well as their differential associations with PTSD symptom severity and related peri- and post-assault characteristics.	Participants were drawn from a large, multisite (i.e., Lincoln and Mississippi; Oxford, Ohio) study of sexual revictimization among 490 young adult women (aged 18–25 years) in the community. Participants included in the present study were 161 women who reported a completed rape since the age of 18 years. (mean age: 22.24). Ethnically/racially diverse (69.4% White; 28.5% African American/Black; 4.6% Asian/Pacific Islander). Participants unmarried (83.2%) and had no children (74.5%). 49.7% were full-time or part-time students. 77.6% self-identified as heterosexual, 13% identifying as bisexual, 5.6% as gay/lesbian, and 3.1% questioning.	Sexual Experiences Survey (SES). List of peritraumatic emotions and asked to indicate on a scale (from 1 to 7) the degree to which they experienced each emotion at the time of the unwanted sexual activity. PTSD Checklist–Civilian Version (PCL-C).	Combined type rapes were associated with significantly more severe PTSD symptoms than forcible-only, and impaired type rapes. Differences were also found for peritraumatic fear and injury, and rape acknowledgment.
Study 12. Messman-Moore et al. (2015)	Cross-sectional and longitudinal (n.s.)	To examine emotion dysregulation, coping drinking motives, and alcohol-related problems as predictors, and consequences of alcohol involved sexual assault.	424 female undergraduates at a midsized (mean age = 9.78) (19.4% first-year, 22.3% sophomores, 22.1% juniors and 27.4% seniors). Public, rural university in the Midwest of the US. 88.4% Caucasian, 26% were members of a sorority, 98.3% were unmarried, 72.4% were sexually active, and 48.6% being in an exclusive romantic relationship. 46.2% income greater than $100,000.	23-item Rutgers Alcohol Problem Index (RAPI). Coping subscale of the Drinking Motives Questionnaire–Revised (DMQ-R). Difficulties in Emotion Regulation Scale (DERS). Revised Sexual Experiences Survey (AISA).	In the short-term AISA is predicted by drinking to cope and emotion dysregulation. Alcohol problems increased risk for AISA in the long term, and AISA history predicted AISA revictimization over time. Drinking to cope and alcohol-related problems predicted future victimization, and their effects appear to vary over time. Coping drinking motives were a predictor, and consequence of AISA, suggesting a cyclical pattern.
Study 13. Nelson and Fischer (2021)	Longitudinal	To investigate the impact of a recent sexual assault on the endorsement of coping motives for drinking in first year college women.	104 college women at a large Southeastern university, ranged in age from 17 to 22 years old with a modal age of 18 years (*M* = 18.04, SD = .30). 84% of the sample identified as heterosexual, while 1% identified as homosexual, and 8.3% identified as bisexual. 14% participants did not report their sexuality. 75, 5% of participants identified themselves as White, 17.0% as Black, 3.8% as Asian American, 1.9% as Hispanic, 0.9% as biracial, and 0.9% as another ethnicity. 2 participants did not report their ethnicity.	Participants were asked to identify their age, year in college, ethnicity, biological sex, and sexual orientation. Sexual Experiences Survey (SES); Drinking Motives Questionnaire-Revised (DMQ); Timeline Followback for Drinking (TLFB).	Sexual assault during the first semester of college was found to be a significant predictor of using alcohol as a coping motives at the end of the semester after controlling for lifetime history of sexual assault, and endorsement of coping motives at Time 1.
Study 14. Pegram and Abbey (2019)	Cross-sectional (n.s)	(1) To examine psychological and physical health outcomes associated with sexual assault victimization in a community sample of African American and Caucasian survivors. (2) To examine similarities and differences in the relationships between sexual assault severity and health for African American and Caucasian survivors.	Women who experienced a sexual assault since age 14. Of the 221 participants, 54.8% identified as African American and 45.2% identified as Caucasian. Women aged between 18 and 49 years (mean age: 31.84 years). 93% of participants had at least a high school degree. Recruited: random digit dialing to identify eligible participants in the metropolitan Detroit area (US).	Modified version of the Sexual Experiences Survey (SES); Participants answered a detailed list of questions about one sexual assault incident: based on the assault type, recency of the event. Severity of the incident was assessed using a combination of 7 different indicators: a checklist of 14 tactics to assess force; 8 items checklist to assess injuries; checklist of 5 threatening actions to assess post-assault threat; checklist of 20 items to assess immediate negative affect. Participants were asked questions to assess: what was considered a sexual offense, the perceived seriousness at the time and seriousness now, and which the incident disrupted their relationships with men. Davidson’ Trauma Scale; abbreviated version of Beck’s Depression Inventory (Beck, 1967); Hilton’s (1987) 13-item Problematic Drinking subscale.	23% of African Americans, and 26% of Caucasians describing a completed rape, 7% of African Americans, and 7% of Caucasians describing an attempted rape, 32% of African Americans, and 35% of Caucasians describing verbally coerced penetrative sex, and 37% of African Americans, and 32% of Caucasians describing forced sexual contact (χ^2^ = .75, *p* = .86). There was only one significant difference. Controlling for income, Caucasians reported more depressive symptoms (*M* = 7.34, SD = 8.41) than did African Americans (*M* = 3.93, SD = 6.09), *F*(1, 219) *=* 12.14, *p* = .001. For African American and Caucasian women, assault severity was significantly positively associated with PTSD symptoms, and depressive symptoms were significantly positively associated with physical health symptoms. Among African American survivors, sexual assault severity affected physical health symptoms indirectly through its impact on depressive symptoms, and assault severity indirectly affected drinking problems through its impact on PTSD symptoms.
Study 15. Petrak et al. (1997)	Cross-sectional (n.s.)	To describe the nature of the sexual assaults, and subsequent psychological difficulties in women referred to clinical psychology in GUM setting.	32 women victims of sexual assault referred over a one year period to clinical psychology in an East London Genitourinary medicine (GUM) clinic. Women’s mean age was 25.7 years (SD 7.5), ranging from 15 to 42 years. Among the participants, 59.4% were Caucasian, 28.1% Black Afro-Caribbean, 9.4% South Asian, and 3.1% belonged to other ethnicities.	Data were collected by pro forma on demographic factors, previous assault and psychiatric history, characteristics of the sexual assault, outcome, and psychological presentation. The therapist was required to indicate the absence or presence of the symptom as observed on assessment.	Psychological difficulties reported by the clinical psychologist in women attending therapy (*n* = 23): depressed/low mood (95.7%); anxiety (91.3%); tension/irritability; tearfulness; intrusive images/thoughts of the assault; sleep disturbance; avoiding places/things reminding of the assault; increased arousability; concentration difficulties; nightmares; self-blame/guilt; self-disgust; ongoing fear of assailant; anger; relationship difficulties; suicidal thoughts; sexual difficulties; using more alcohol or drugs. Other issues that were confusing was: sexual identity, moving home, changing jobs or not feeling capable to work. Also, they expressed fears about the consequences of the assault (e.g., going to the police, and to court, pregnancy, sexually transmitted infections including HIV). Some women, for example, were concern to the ‘marriagability’ of a person who had been raped.
Study 16. Pohane et al. (2020)	Cross-sectional	To study the psychopathology, perceived social support, and coping mechanism in ASA survivors in urban center India.	50 women survivors of ASA. 29 of the participants were younger than 25 years (mean age: 25.6 ± 7.2 years). 62% of the participants were never married, and 70% were educated not more than 12th standard. 30 of the participants belonged to lower socioeconomic status, 14 were from middle and 6 upper socioeconomic status. Participants were recruited from the psychiatry department of the institute.	Mini-International Neuropsychiatric Interview (MINI); Multidimensional Scale for Perceived Social Support (MSPSS); Mechanism of Coping Scale (MOCS).	Psychiatric morbidity was present in 82% participants, with 60% diagnosed as major depressive disorder (MDD), 12% suffered from PTSD and 10% had both. Suicidal ideas were reported by 66% participants.
Study 17. Rhew et al. (2017)	Longitudinal	To examine effects of sexual assault victimization on later typical alcohol use, and alcohol-related consequences among young sexual minority women.	A total of 4119 women completed the online screening survey. Information was collected over four annual assessments from a national sample longitudinal study of young adult SMW’s health and health behaviors. Eligible women (*n* = 1877) were invited to participate in the study. 1057 residing in the US were retained in the study. Participants identified as lesbian or bisexual and were 18 to 25 years old at baseline. Race/ethnicity: white, non-Hispanic (63.4%); African American, non-Hispanic (8.5%); Asian American, non-Hispanic (2.5%); multiracial, non-Hispanic (14.3%); another race, non-Hispanic (2.2%); Hispanic, any race (11.3%).	Revised Sexual Experiences Survey (SES); Daily Drinking Questionnaire (DDQ); Young Adult Alcohol Consequences Questionnaire (YAACQ); PTSD Checklist (PCL); CES-D; (GAD-7); Daily Heterosexist Experiences Questionnaire (DHQ); Traumatic Life Events Questionnaire (TLEQ); modified version of Drinking Norms Rating Form.	Severe sexual assault at the prior assessment was associated with a 71% higher number of typical weekly drinks (Count Ratio [CR] = 1.71; 95% confidence interval [CI]: 1.27, 2.31), and 63% higher number of alcohol-related consequences (CR = 1.63; 95% CI: 1.21, 2.20). There was also a strong association between 2-year cumulative sexual assault severity and drinking-related consequences (CR = 1.27; 95% CI: 1.12, 1.43).
Study 18. Rothman et al. (2021)	Longitudinal, repeated-measures	To examine immediate, and long-term consequences of college sexual assault (C-SA) among women with no prior sexual assault history.	Participants were 404 women from US with (*n* = 201) and without (*n* = 203) C-SA history between the ages of 28 and 30 and have at least a bachelor’s degree. They were recruited from throughout the United States through the Amazon Mechanical Turk subject pool. Participants were 33.9% Caucasian, 32.7% Black, and 33.4% Hispanic; similarly, 48.9% of the Caucasian, 49.2% of the Black, and 51.1% of the Hispanic women experienced C-SA. Within the sexual assault sample, on average, women participated in this study 8.74 years (*Mdn* = 8.395 years) post-C-SA. There were no differences in employment [χ^2^(4) = 3.26, *p* = .515], income [χ^2^(6) = 9.22, *p* = .161], or education [χ^2^(2) = 2.08, *p* = .352] between woman with and without a C-SA history. Furthermore, there were no differences in employment [χ^2^(8) = 7.27, *p* = .507], income [χ^2^(12) = 10.81, *p* = .544], or education [χ^2^(4) = 4.67, *p* = .322] by race or ethnicity.	Information about the timing of the C-SA relative to the academic year of college; Post-Traumatic Stress Disorder–8 Items (PTSD-8); 10-item Center for Epidemiologic Studies–Depression scale (CES-D); 7 item measure of Generalized Anxiety Disorder symptoms (GAD-7); 4 item Couples Satisfaction Index (CSI-4); 2 subscales (6 items each) of the Personal Assessment of Intimacy in Relationship (PAIR), 3 items corresponding to DSM-IV-TR substance abuse diagnostic criteria.	There was a significant group-by-time interaction for GPA [(*F*(1402) = 27.13, *p* < .001], days of missed school due to substance use [(*F*(1402) = 6.59, *p* = .011], and number of romantic relationships [(*F*(1402) = 24.14, *p* < .001] following the assault. Furthermore, women who experienced C-SA compared to those that did not, had significantly greater PTS symptoms (*b* = 4.48, SE = 0.63, *t =* 7.71, *p* < .001), depression (*b* = 3.46, SE = 0.64, *t =* 5.35, *p* < .001), and anxiety symptoms (*b* = 2.46, SE = .55, *t =* 4.61, *p* < .001) about 9 years post-assault.
Study 19. Stein et al. (2004)	Cross-sectional	To determine whether there is an association between sexual assault history and measures of somatic symptoms and illness attitudes.	219 women in a Veteran’s Affairs primary care outpatient clinic from VA San Diego Health-care System (VASDHS). The ethnic groups of the participants with a history of sexual assault were: 23% African American, 68% Caucasian, and 9% from other ethnic groups. Among those without a history of sexual assault, there were 12% African American, 76% Caucasian, and 12% from other ethnic groups.	Questionnaire about sociodemographic characteristics Clinician-Administered PTSD for Diagnostic and Statistical Manual of Mental Disorders (4th ed.). To measure somatization, somatization subscale of the Symptom Checklist 90-Revised (SCL-90-R) was used. Health anxiety was measure using 4 items taken from the Illness Attitudes Scale.	97 women (43.9%) reported experience(s) of sexual assault. Sexual assault was associated with a significant increment in: somatization scores, physical complaints across multiple symptom domains, and health anxiety (illness fear or illness worry). They experimented numerous days being ill in the prior 6 months, and visiting often primary care resources in the prior 6 months
Study 20. Vidal and Petrak (2007)	Cross-sectional (n.s.)	To assess feelings of shame in women survivors of adult sexual assault by using a series of self-rating scales.	A group of 25 female survivors of adult sexual assault from London. Most of participants identifying themselves as belonging to a minority ethnic group (*n* = 15). Participants were recruited from a sexual health service and via media advertisement within East London.	Questions of demographic information. Sexual assault characteristics and questions about the most recent experience of ASA. Participants answered detailed questions to assess the impact of the assault and shame response. The Experience of Shame Scale (ESS); the Impact of Events Scale–Revised (IES-R).	17 participants experienced physical consequences following the assault (e.g., pelvic pain, pregnancy and STIs). 20 women had at some point avoided telling others; 13 had kept the assault a secret; 16 felt they were to blame for the assault. In relation to the IES-R, scores from 22 of the women suggested the presence of traumatic stress. Shame was a noteworthy psychological response for this group of women (up to 75% felt ashamed about themselves following sexual assault). It were found significantly: a history of previous sexual victimization, physical consequences, self-blame, concealing the assault and being assaulted by a known assailant. Results also indicated a significant relationship between shame and traumatic stress.
Study 21. Yeh et al. (2021)	Longitudinal with control-group (n.s.)	To examine subjective and objective sleep quality in young women with PTSD following sexual assault compared with a control group at baseline and after one year of treatment.	74 women with PTSD following sexual assault (18–45 years) with a history of Sexual Assault in the 1 to 6 months before study enrollment) and 64 healthy controls with no history of sexual assault (18–45 years with no PTSD or history of sexual assault). 44.6% of the PTSD group were Caucasian, and 55.4% African Brazilian. Control group: 71.9% Caucasian and 28.1% African Brazilian. Recruited from: the largest women´s public health facility in Sāo Paulo (Brazil).	The Clinician-Administered PTSD Scale for DSM-5 (CAPS-5); Mini-International Neuropsychiatric Interview (MINI); Beck Depression Inventory–2º Edition (BDI-II); Beck Anxiety Inventory (BAI); Pittsburgh Sleep Quality Index (PSQI); Pittsburgh Sleep Quality Index addendum for PTSD (PSQI-A); Epworth Sleepiness Scale (ESS); Insomnia Severity Index (ISI); Fatigue impact: Modified Fatigue Impact Scale (MFIS).	The PTSD group had significantly higher scores in the clinical and sleep measurements than control group. Although the PTSD group reported poorer subjective sleep quality than healthy controls, there were few between-group differences in objective sleep.

n.s. (Not specified: we observed the design along the text but it wasn’t specified by authors); PTSD (Posttraumatic Stress Disorder); ASA (Adult Sexual Assault); PEP (Post-Exposure prophylaxis); SDB (Sleep disorder Breathing); PLMD (Periodic Limbic Movement Disorder); HBCUs (Historically Black Colleges or Universities); GPA (Grade Point Average); AISA (Alcohol-Involved Sexual Assault)

The collection and systematization of sociodemographic data varied between studies. Specifically, six studies (Studies 4, 5, 6, 7, 10, and 16) did not provide information on the ethnicity of the women involved. One study had a larger sample of Black women (Study 8), another study had a larger sample of Caucasian women (Study 12), and one study included a sample which identified as belonging to a minority ethnic group (Study 20). The remaining studies (Studies 1, 2, 3, 9, 11, 13, 14, 15, 17, 18, 19, and 21) had mixed racial/ethnic samples, including African American, European American, Latino/Hispanic, African, Caucasian, Asian, multiethnic, and others. To assess the impact of ASA on women, the 21 studies analyzed used various measurement tools. The most commonly used were the Center for Epidemiological Studies Depression Scale (CES-D) to assess depressive symptoms, the Generalized Anxiety Disorder-7 (GAD-7) to assess anxiety symptoms, and the Mini-International Neuropsychiatric Interview (MINI) and the Diagnostic and Statistical Manual of Mental Disorders (DSM) to assess multiple symptoms.

### Depressive Symptoms

To measure the impact of ASA, 13 of the studies assessed depressive symptoms experienced by survivors after the traumatic event. Five of the studies (Studies 1, 8, 9, 17, and 18) used the CES-D to assess depressive symptoms. Three studies (Studies 7, 14, and 21) used the Beck Depression Inventory (BDI). One study (Study 3) used the Patient Health Questionnaire-9 (PHQ-9), and Study 16 used the MINI. Study 10 assessed symptomatology using the DSM-III, Study 6 used the Florence Psychiatric Interview, and in Study 15, depression symptoms were observed by the therapist. Eight studies found that women victims suffered from depressive symptoms after the assault (Studies 1, 3, 6, 8, 10, 14, 15, and 16). In Study 1, 84.3% of a sample of 140 participants had a depression symptom score of 16 or higher. Study 3 found that women who experienced first-semester sexual assault were approximately 2.5 times more likely to have significant depression symptoms (OR = 2.45) compared to women who were not assaulted in their first semester. In Study 6, 75% of the 40 adult women victims of rape were diagnosed with major depression. Study 8 reported that 53% of survivors of incapacitated sexual assault and 50% of survivors of physically forced sexual assault had mean depressive symptom scores of 16 (compared to 12.5 among women who had not been assaulted). Study 10 found that 85.29% of participants showed depressive symptoms immediately after the rape, with 76.47% showing depression in the long term. Study 14 did not provide percentages on depressive symptoms but indicated higher levels of depression in Caucasian women compared to African American women, controlling for income. Study 15 found that 95.7% of a sample of 32 female victims of sexual assault attending therapy showed depression/low mood. Study 16 found that 60% of a sample of 50 female ASA survivors were diagnosed with major depressive disorder (MDD). In general, between 60% and 95.7% of the women victims of ASA in the studies reviewed experienced some type of depressive symptoms or disorder.

Study 7 also highlighted the co-occurrence of PTSD and depression, indicating comorbidity. Additionally, three studies found differences in depressive symptoms between women of different races. Study 14 showed that, controlled by income, Caucasian women reported more depressive symptoms (*M* = 7.34, SD = 8.41) than African American women (*M* = 3.93, SD = 6.09, *F*(1, 219) = 12.14, *p* = .001). For both African American and Caucasian women, depressive symptoms were significantly positively associated with physical health symptoms. Study 9 found that for African American survivors, the severity of sexual assault indirectly influenced physical health symptoms through its effect on depressive symptoms. Depression was also examined as a mediator of the relationship between sexual assault and health risk behaviors, with evidence of moderated mediation for European American women but not for African American, Latina, and Asian American women. Study 8 also examined depressive symptoms as a mediator between sexual assault and health risks (hazardous drinking and engaging in sexual behavior to regulate negative affect), with evidence of moderated mediation for European American women but not for African American, Latin American, and Asian American women. Moreover, a study of women attending therapy (*n* = 23) revealed that 95.7% exhibited depressive symptoms, documenting the frequency of psychological difficulties experienced by women seeking therapy after ASA (Study 15). Correlation analyses confirmed strong relationships between PTSD and other depression symptoms (Study 7). Finally, Study 21 found that women who had experienced sexual assault had significantly higher levels of depression, even after controlling for depressive symptoms. When comparing the symptoms of sexually assaulted university women with a control group, significantly greater depressive symptoms were observed (*b* = 3.46, SE = 0.64, *t* = 5.35, *p* < .001) (Study 18). Study 18 conducted a series of 2 × 2 repeated measures analysis of variance (ANOVAs) for group comparisons. The data show that experiencing ASA predicts the manifestation of psychological symptoms. The document does not provide information on the effect size.

### Anxiety Symptoms

Eleven of the 21 studies measured anxiety symptoms in female victims to analyze the impact of the traumatic event. A variety of measures were used. Specifically, the GAD-7 was employed in three studies (Studies 3, 17, and 18). In Study 15, anxiety symptoms were observed by the therapist. The remaining studies used different measures: Spanish Survey of Violence Against Women (Study 4); Florence Psychiatric Interview (Study 6); Beck Anxiety Inventory (BAI) (Studies 7 and 21); Four-Dimensional Anxiety Scale (FDAS) (Study 9); MINI (Study 16); and Administered PTSD (Study 19). All studies indicated that women who experienced sexual assault developed anxiety or generalized anxiety symptoms (Studies 3, 4, 6, 7, 9, 15, 17, 18, 19, and 21), even years later (Study 18). Specifically, around 91% of women who attended therapy after being assaulted presented anxiety symptoms (Study 15). Moreover, Study 7's correlation analyses confirmed strong relationships between PTSD and other anxiety symptoms. Study 8 examined anxiety as a mediator between sexual assault and health risks (hazardous drinking and engaging in sexual behavior to regulate negative affect), with evidence of moderated mediation for European American women but not for African American, Latin American, and Asian American women. Additionally, in Studies 17 and 21, anxiety symptoms were used as a covariate to measure drinking problems and sleep dysfunction.

### PTSD Symptoms

More than half of the studies (*n* = 11) included PTSD symptoms to analyze the consequences of sexual aggression in women (Studies 5, 6, 7, 8, 11, 14, 16, 17, 18, 20, and 21). Several measures were used to assess PTSD symptoms: Impact of Event Scale (IES) (Study 5); Florence Psychiatric Interview (Study 6); Traumatic Stress Symptom Checklist (TSSC) (Study 7); a slightly modified version of the Primary Care PTSD (PC-PTSD) screen (Study 8); PTSD Checklist–Civilian Version (PCL-C) (Study 11); PTSD Checklist (PCL) (Study 17); Davison Trauma Scale (Study 14); MINI (Study 16); Post-Traumatic Stress Disorder–8 Items (PTSD-8) (Study 18); Impact of Events Scale–Revised (IES-R) (Study 20); and the Clinician-Administered PTSD Scale for DSM-5 (CAPS-5) (Study 21). Women who experienced sexual assault showed a significantly higher prevalence of PTSD (Studies 5, 6, 7, 8, 16, and 21), even 9 years after the assault (Study 18). One study found that 68% of women who were sexually assaulted suffered from PTSD, and those with PTSD were more likely to have a lower socioeconomic status and were nearly seven times more likely to experience suicidal thoughts (Study 7). Nine studies compared groups of female victims with control groups. In studies comparing PTSD symptoms alone, survivors of sexual assault were more likely to screen positive for PTSD than non-victims (Studies 5, 6, 8, 14, and 18).

Another consequence shown in two studies was that the PTSD group had worse subjective sleep quality (Study 21) and more sleep disorders (Study 5) than the control group. Furthermore, Study 14 demonstrated that the severity of the assault indirectly affected drinking problems as a coping mechanism for PTSD symptoms in Afro-descendant women. Similarly, one study used PTSD symptoms as a covariate to measure drinking problems, showing a significantly higher proportion of participants with PTSD compared to the general population (Study 17). Moreover, the severity of the assault was associated with more severe PTSD symptoms (Study 14). For example, combined-type rapes (characterized by both force and substance involvement) were linked to significantly more severe PTSD symptoms (Study 11), and survivors who reported sustaining an injury during the assault were significantly more likely to screen positive for PTSD than those who did not sustain an injury (Study 8).

### Alcohol Use

Approximately 30% of the studies (*n* = 6) analyzed alcohol use as a consequence of sexual assault among female victims (Studies 2, 12, 13, 14, 17, and 18). In Study 2, alcohol use was assessed using several instruments: Quantity Frequency Questionnaire (QF), Information to compute blood alcohol content (BAC), a modified version of the Daily Drinking Questionnaire (DDQ), Comprehensive Effects of Alcohol Questionnaire (CEOA), and a modified version of the Rutgers Alcohol Problem Index (RAPI). Study 12 used the 23-item RAPI and the Drinking Motives Questionnaire–Revised (DMQ-R). Study 13 used DMQ-R and Timeline Followback for Drinking (TLFB). Study 14 utilized a 13-item Problematic Drinking subscale. Finally, Study 17 used SES, Daily Drinking Questionnaire (DDQ), Young Adult Alcohol Consequences Questionnaire (YAACQ), and a modified version of the Drinking Norms Rating Form. Study 18 employed three items corresponding to DSM-IV-TR substance abuse diagnostic criteria.

Women with a history of sexual assault reported greater alcohol use than women without a trauma history (Study 2). Additionally, women with an Alcohol-Involved Assault (AIA) reported greater alcohol use and positive alcohol expectancies than women without a trauma history and those with a non-alcohol-involved assault (Study 2). Drinking to cope was both a predictor and a consequence of Alcohol-Involved Sexual Assault (AISA) (Study 12). Drinking to cope and emotion dysregulation also predicted AISA in the short term. Long-term alcohol problems increased the risk of AISA, and a history of AISA predicted AISA revictimization (Study 12). The severity of sexual assault indirectly influenced alcohol problems through its effect on PTSD symptoms (Study 14). Furthermore, the severity of sexual assault impacted alcohol-related consequences, being associated with a higher number of drinks per week (73%) and consequences of alcohol abuse (63%). Long-term habitual drinking showed differences in alcohol-related consequences among those who have been sexually assaulted, as well as among regular drinkers, although the association was smaller for regular drinkers compared to assault victims (Study 17). Exposure to sexual assault during the first semester of college was linked to an increased likelihood of using alcohol as a coping mechanism by the end of the semester, even when controlling for prior sexual assault history and initial endorsement of coping motives (Study 13). These findings reflect the impact of sexual assault on the development of alcohol dependence (Studies 2, 12, 13, 14, 17, and 18).

### Sleep Disorders

Three studies investigated the impact of sexual assault on sleep quality in female victims (Studies 5, 15, and 21). To assess sleep disorders, Study 5 used the Pittsburgh Sleep Quality Index (PSQI). In Study 15, the therapist observed symptoms, and Study 21 used the PSQI-A, Insomnia Severity Index (ISI), and the Modified Fatigue Impact Scale (MFIS). Study 5 assessed the causal influence of exposed and unexposed groups, comparing sleep-related events among women living in Bogotá. This study found that sleep disorders were significantly more prevalent in women who had been raped compared to the unexposed group. Problems identified included sleepiness (35.29%), sleep disturbances (52.94%), trouble sleeping due to needing to get up to go to the bathroom (26.47%), difficulty breathing (29.41%), loud coughing or snoring (26.47%), and daytime dysfunction (55.88%). Study 15 highlighted psychological difficulties reported and observed by the clinical psychologist, including sleep disturbances and nightmares. Finally, Study 21 indicated that the PTSD group (survivors of sexual assault) reported poorer subjective sleep quality and higher sleep disturbance scores than women who had not been assaulted.

### Other Consequences

In addition to the specific consequences discussed above, nine studies reflected on various other impacts on women survivors of sexual assault (Studies 4, 6, 7, 10, 14, 15, 18, 19, and 20). These consequences were measured using a variety of tools, including the Spanish Survey of Violence Against Women (Study 4), Florence Psychiatric Interview (Study 6), The Medical Outcomes Study Short Form-36 (SF-36) (Study 7), structured clinical interview for DSM-III (Study 10), SES (Study 14), therapist-observed symptomatology (Study 15), Couples Satisfaction Index (CSI-4) and Personal Assessment of Intimacy in Relationship (PAIR) (Study 18), the Somatization subscale of the Symptom Checklist 90-Revised (SCL-90-R) (Study 19), and the Experience of Shame Scale (ESS) and Impact of Events Scale-Revised (IES-R) (Study 20). Some of the consequences found included feelings of worthlessness, sadness, a desire to cry without reason (Study 4); avoidance of thoughts or conversations about the trauma, suicidal ideation, and social phobia (Study 10); irritability, tearfulness, intrusive images or thoughts of the assault, avoiding places or things reminding of the assault, increased arousability, and concentration difficulties (Study 15); days missed from college due to substance use (Study 18); and relationship difficulties following the assault (Studies 15 and 18). Additionally, having an abortion was strongly predicted by suicidal thoughts and impulses (Study 10). The consequences of ASA can also negatively impact the physiological health of victims, including sexual disorders, dysfunction, or difficulties (Studies 6, 10, and 15), a significant increase in somatization scores, physical complaints across multiple symptom domains, health anxiety, frequent sick days, and numerous visits to primary care resources in the six months before the study (Study 19). Women with PTSD were significantly more likely to report other health problems, including poor general health, low vitality, impaired social and physical function, and impaired physical and emotional roles (Study 7). Furthermore, Study 15 found confusion about sexual identity (not defined in the text), moving homes, changing jobs, or not feeling capable of working. Among African American survivors, the severity of sexual assault indirectly influenced physical health symptoms through its effect on depressive symptoms (Study 14). Cultural issues, such as concerns about the “marriageability” of a person who had been raped, were also significant for some women (Study 15).

It is also important to note that the consequences of ASA can have a negative impact on the physiological health of women victims. This can include a variety of problems, such as sexual disorder, dysfunction, or difficulties (Study 6, Study 10, and Study 15), a significant increase in somatization scores, physical complaints across multiple symptom domains, and health anxiety, experiencing numerous sick days and frequent visits to primary care resources in the 6 months prior to the study (Study 19). The women victims of ASA presenting with PTSD were significantly more likely than those without to have scale scores indicative of other health problems including sense of poor general health, low vitality, impaired social and physical function, and impaired physical and emotional role (Study 7). Moreover, Study 15 found also that there was a confusion about sexual identity (not defined in the text), moving home, changing jobs or not feeling capable to work. Among African American survivors, the severity of sexual assault indirectly influenced physical health symptoms through its effect on depressive symptoms (Study 14). Cultural issues were a particular concern to some women, for example, the “marriageability” of a person who had been raped (Study 15).

## Discussion

Research indicates that being a woman significantly increases the likelihood of being a victim of sexual assault (Cruz, 2017). Approximately 15% to 25% of women have experienced sexual assault in their lifetime (Campbell & Wasco, 2005; Vickerman & Margolin, 2009). For example, Study 3 highlights the magnitude of this problem, showing that 28% of a sample of 483 female first-year college students reported being victims of sexual assault, with 12% experiencing at least one incident involving threat, force, or incapacitation during their first semester. Study 9 reveals that between 12% and 21% of university women from different ethnic backgrounds (European American, Latina, African American, and Asian American) have suffered some form of sexual assault in adolescence or adulthood. This scoping review provides evidence that being a victim of sexual assault (ASA) has significant consequences on the health of women, affecting their psychological and social well-being. The findings are consistent with those of other studies (Arboleda et al., 2011; Campbell et al., 2009; Campbell & Wasco, 2005; Cruz, 2017; Echeburúa & Guerricaechevarría, 2011; Echeburúa et al., 2013; Koss et al., 2003; Kilpatrick et al., 2007; Luce et al., 2010; Perilloux et al., 2012; Pico-Alfonso et al., 2008; Rellini, 2008; Vickerman & Margolin, 2009). Studies comparing women with and without a history of ASA showed that victims experience significant psychosocial impacts, with various negative consequences affecting their daily lives (Blais & Monteith, 2019; Campbell et al., 2009; Echeburúa et al., 2013; Jonker et al., 2018; van Berlo & Ensink, 2000) (Studies 2, 5, 6, and 21).

Being a victim of violence has emotional consequences for those affected (Arboleda et al., 2011; Echeburúa & Guerricaechevarría, 2011; Pico-Alfonso et al., 2008). Approximately 25% of victims of any violent behavior develop PTSD, but this rises to 50% to 60% for women who have been sexually assaulted (Echeburúa et al., 1997, 2013). Consistent with previous studies on sexual assault (Blais & Monteith, 2019; Campbell et al., 2009; Kilpatrick et al., 2007; Koss et al., 2003), it was shown that women survivors of ASA were more likely to develop PTSD than women who were not exposed to such traumatic events (Studies 5, 6, 7, 8, 16, 18, and 20). Additionally, suffering from PTSD impacts survivors' quality of life and other areas of their daily lives, such as sleep quality (Ullman et al., 2007; Wadsworth et al., 2018) (Studies 5, 15, and 21), or developing drinking problems to alleviate symptoms (Wadsworth et al., 2018) (Studies 13 and 14).

In addition to PTSD, ASA victims may experience emotional distress that negatively impacts their physical health. This is supported by several studies. Study 7 recorded a range of other health problems, including a sense of poor general health, low vitality, impaired social and physical function, and impaired physical and emotional roles. Study 18 found that ASA victims had higher somatization scores and more physical complaints than those who had not been sexually assaulted. Survivors of ASA may also experience psychological sequelae such as anxiety, fear, emotional lability, disbelief, and guilt (Luce et al., 2010). This review shows that women survivors develop depressive symptoms or disorders (Blais & Monteith, 2019; Campbell et al., 2009; Cruz, 2017; Jonker et al., 2018; Koss et al., 2003; Newins et al., 2021) (Studies 1, 3, 6, 7, 8, 9, 10, 14, 15, 16, 17, 18, and 21); anxiety symptoms or generalized anxiety symptoms (Echeburúa et al., 2013) (Studies 3, 4, 6, 7, 9, 15, 17, 19, and 21), even years after the rape (Study 18); PTSD (Echeburúa et al., 1997) (Studies 5, 6, 7, 8, 16, and 21); drinking problems (Newins et al., 2021) (Studies 2, 12, 13, 14, and 17); and sleep disorders (Studies 5, 15, and 21).

Regarding studies that report depressive symptoms or disorders, the variety of instruments used (e.g., CES-D scale, or BDI) means that comparisons of results must be made with caution. Some studies show retrospective or cross-sectional data (e.g., Studies 1, 6, and 10), which entails certain limitations due to potential recall bias, difficulty in measuring confounding variables, or the impossibility of establishing directionality between variables. Additionally, some studies (e.g., Studies 1, 6, 10, and 15) recruited samples from clinical centers or survivors attending therapy, which may result in a bias in the symptomatology identified. These considerations also apply to other types of symptoms addressed in this review.

Other consequences have also been observed, such as suicidal thoughts (Ullman, 2004) (Studies 7 and 15), social phobia (Ramos-Lira et al., 2001) (Study 10), sadness (Study 4), shame (Study 20), differences in the number of romantic relationships after the assault (Studies 15 and 18), substance abuse (Ramos-Lira et al., 2001) (Study 15), and less frequent attendance at college due to substance use (Study 18). Beyond psychological consequences, being a victim of ASA significantly impacts other areas of women’s lives, affecting their sexual health and functioning (O’Callaghan et al., 2019; Perilloux et al., 2012; Rellini, 2008), leading to the development of sexual avoidance behaviors (Echeburúa et al., 2013; O’Callaghan et al., 2019) and sexual dysfunction (Mohammed & Hashish, 2015) (Studies 6, 15, and 19). Sexual behavior has also been found to be used as an affect regulation strategy to mediate the relationship between sexual assault and depression and anxiety (Study 9). Sexual assault can impact women's physical health and daily living activities, increasing the risk of repeated sexual victimization (Kilpatrick et al., 2007; Koss et al., 2003).

Social determinants of health affecting women included in the studies also influence the consequences of sexual assault and may even exacerbate them (Studies 1, 7, 9, and 14). Income, for instance, is associated with access to various coping resources that might mitigate the impact of sexual assault severity (Study 14). Cultural differences also play a role in how women conceptualize these violent experiences and how society addresses them, such as victim-blaming (Studies 1 and 9). One key factor influencing recovery is perceived social support (Study 7).

Thus, the impact of ASA on women is shaped by multiple factors, including victim characteristics, sociocultural norms, the nature of the aggression, the context in which it occurs, and the help-seeking behavior of victims. For example, rape in the context of war exacerbates the traumatic experience due to its systematic occurrence and the oppressive conditions faced by women, coupled with a lack of resources to repair the damage (e.g., inadequate hospital care, psychiatric and psychological care, prophylaxis) (Study 10). Negative experiences when seeking help (informal or formal) for a previous assault can delay further help-seeking and exacerbate psychological distress (Campbell et al., 2009) (Studies 7 and 15). These factors influence the impact of the assault on women’s psychological well-being (Echeburúa et al., 1997) (Study 20) and the coping strategies they develop. This review has shown that ASA can affect women of all ethnicities, races, socioeconomic statuses, and educational levels. It is necessary to attend to the different manifestations that may occur, applying an intersectional approach, in order to respond in the most appropriate way in each case (Studies 1, 7, 9, 10, 14, 15, 17, and 20). This idea is consistent with other studies (Campbell et al., 2009; Perilloux et al., 2012).

### Strengths and Limitations

The review process has several limitations. First, the systematized search was conducted only in English and Spanish, limiting the possibility of finding relevant studies in other languages. Second, the variety of terminology used to describe sample profiles poses a limitation for comparing study results. Third, the age of participants is reported inconsistently across studies, sometimes as a mean and sometimes as a standard deviation. Fourth, the instruments used to measure the same variables vary, making it difficult to compare results between studies. Fifth, different terms for sexual assault, such as “sexual aggression,” “sexual harassment,” “rape,” or “sexual abuse,” complicate the analyses. Sixth, the design of some studies is not clearly specified, and cross-sectional studies limit the understanding of the evolution of sexual assault consequences and the relationships between variables. Lastly, the data on sexual assaults were collected in diverse contexts (e.g., clinical centers, universities, war zones), which may influence the impact on victims but was not analyzed. One of the strengths of this work is that it analyzes the consequences of ASA on various aspects of women's lives, including those that may not meet clinical diagnostic thresholds for mental health disorders. Second, this review updates the existing knowledge about the consequences of ASA. Third, despite its relevance, ASA has been infrequently explored due to difficulties in identifying symptoms and relating them to sexual assault, the challenge of disclosure, and the potential for secondary victimization. Finally, this scoping review identifies new areas of work in the field, such as the need for a multilevel approach and an intersectional and feminist perspective. This approach considers the subordination women experience at different ecological levels and its influence on the consequences of sexual assault.

### Conclusions

Being a woman increases the risk of ASA, a social problem with significant psychosocial consequences. These include depressive symptoms, anxiety symptoms, PTSD, alcohol dependence, and sleep disorders. Other consequences include sexual dysfunction, increased somatization scores, physical complaints, frequent sick days and primary care visits, feelings of worthlessness, a desire to cry for no reason, avoidance of trauma-related thoughts or conversations, suicidal ideation, social phobia, reduced university attendance due to substance use, and fewer serious romantic relationships post-assault.

Qualitative research is needed to capture the voices of assaulted women and understand how they define their experiences, the implications, and their strengths. More research is also needed on the consequences of ASA, considering women's diversity. An intersectional approach is necessary to enhance the understanding of the impact of ASA at each stage of women's lives.

In summary, understanding the mediating variables between sexual assault and its negative consequences, as well as the multiple impacts of such aggression, will contribute to developing effective interventions. These can help improve the well-being of assaulted women, aiding them in overcoming ASA consequences and politicizing the experience from a feminist perspective.

## Supplementary Information

Below is the link to the electronic supplementary material.

Supplementary file1 (DOCX 43 KB)

## Footnotes

1 Incidents of sexual assault experienced at 14 years of age or older, which is the age considered in literature. Different examples can be found in the articles analyzed: Bedard-Gilligan et al. (2011); Domenech del Río and Sirven (2017); Littleton et al. (2013); Loncar et al. (2006); Pegram and Abbey (2019); Petrak et al. (1997); Vidal and Petrak (2007).

## References

[CR1] Abrahams, N., Jewkes, R., & Mathews, S. (2013). Depressive symptoms after a sexual assault among women: Understanding victim-perpetrator relationships and the role of social perceptions. *African Journal of Psychiatry,**16*(4), 288–293. 10.4314/ajpsy.v16i4.39 24051569 10.4314/ajpsy.v16i4.39

[CR2] American Psychiatric Association. (2013). *Diagnostic and statistical manual of mental disorders* (5th ed.). American Psychiatric Publishing.

[CR3] Arboleda, M. C. P., Cortés, D. C., & Duarte, J. A. (2011). Consecuencias a largo plazo del abuso sexual infantil: Papel de la naturaleza y continuidad del abuso y del ambiente familiar [Long-term consequences of child sexual abuse: Role of the nature and continuity of abuse and the family environment]. *Behavioral Psychology/psicología Conductual. Revista Internacional De Psicología Clínica y De La Salud,**19*(1), 41–56.

[CR4] Arksey, H., & O’Malley, L. (2005). Scoping studies: Towards a methodological framework. *International Journal of Social Research Methodology: Theory & Practice,**8*(1), 19–32. 10.1080/1364557032000119616

[CR5] Bedard-Gilligan, M., Kaysen, D., Desai, S., & Lee, C. M. (2011). Alcohol-involved assault: Associations with posttrauma alcohol use, consequences, and expectancies. *Addictive Behaviors,**36*(11), 1076–1082. 10.1016/j.addbeh.2011.07.001 21813246 10.1016/j.addbeh.2011.07.001 PMC3153602

[CR6] Blais, R. K., & Monteith, L. L. (2019). Suicide ideation in female survivors of military sexual trauma: The trauma source matters. *Suicide and Life-Threatening Behavior,**49*(3), 643–652. 10.1111/sltb.12464 29676496 10.1111/sltb.12464

[CR7] Campbell, G., Biscoe, N., Hendrikx, L., Williamson, V., & Murphy, D. (2022). *Evidence-based treatments for the psychological sequelae of sexual trauma in a female serving military and veteran population: A systematic review*. University of York.

[CR8] Campbell, R., Dworkin, E., & Cabral, G. (2009). An ecological model of the impact of sexual assault on women’s mental health. *Trauma, Violence & Abuse,**10*(3), 225–246. 10.1177/1524838009334456 10.1177/1524838009334456 19433406

[CR9] Campbell, R., & Wasco, S. M. (2005). Understanding rape and sexual assault. *Journal of Interpersonal Violence,**20*(1), 127–131. 10.1177/0886260504268604 15618569 10.1177/0886260504268604

[CR10] Carey, K. B., Norris, A. L., Durney, S. E., Shepardson, R. L., & Carey, M. P. (2018). Mental health consequences of sexual assault among first-year college women. *Journal of American College Health,**66*(6), 480–486. 10.1080/07448481.2018.1431915 29405862 10.1080/07448481.2018.1431915 PMC6311089

[CR11] Cruz, A. (2017). *Factores predictores del impacto psicopatológico en víctimas de agresión sexual* [Predictors of psychopathological impact in victims of sexual assault] (Publication Nº T35349). Madrid: Universidad Complutense de Madrid.

[CR12] Domenech del Río, I., & Sirvent, E. (2017). Non-partner sexual violence against women in Spain: Lifetime prevalence, perpetrators and consequences on mental health. *Journal of Public Health,**39*(4), 738–744. 10.1093/pubmed/fdw111 27738127 10.1093/pubmed/fdw111

[CR13] Echeburrúa, E., & Guerricaechevarría, C. (2011). Tratamiento psicológico de las víctimas de abuso sexual infantil intrafamiliar: Un enfoque integrador [Psychological treatment of victims of intrafamilial child sexual abuse: An integrative approach]. *Behavioral Psychology/psicología Conductual. Revista Internacional De Psicología Clínica y De La Salud,**19*(2), 469–486.

[CR14] Echeburúa, E., Corral, P., Amor, P. J., Zubizarreta, I., & Sarasua, B. (1997). Escala de gravedad de síntomas del trastorno de estrés postraumático: Propiedades psicométricas [Post-traumatic stress disorder symptom severity scale: Psychometric properties]. *Análisis y Modificación De Conducta,**23*(90), 503–526.

[CR15] Echeburúa, E., Sarasua, B., Zubizarreta, I., & Corral, P. (2013). Psychological treatment of recent and non-recent adult female victims of sexual assault. *Behavioral Psychology/psicología Conductual. Revista Internacional De Psicología Clínica y De La Salud,**21*(2), 249–269.

[CR16] Escobar-Córdoba, F., & Eslava-Schmalbach, J. (2012). Sleep disorders and posttraumatic stress in raped victims. *Revista De La Facultad De Medicina,**60*(4), 317–324.

[CR17] Faravelli, C., Giugni, A., Salvatori, S., & Ricca, V. (2004). Psychopathology after rape. *American Journal of Psychiatry,**161*(8), 1483–1485. 10.1176/appi.ajp.161.8.1483 15285977 10.1176/appi.ajp.161.8.1483

[CR18] Grotberg, E. H. (1995). *The International Resilience Project: Promoting resilience in children*. Washington, DC: U.S. Department of Education.

[CR19] Jonker, I. E., Lako, D. A. M., Beijersbergen, M. D., Sijbrandij, M., van Hemert, A. M., & Wolf, J. R. L. M. (2018). Factors related to depression and post-traumatic stress disorder in shelter-based abused women. *Violence Against Women,**25*(4), 401–420. 10.1177/1077801218790700 30124130 10.1177/1077801218790700 PMC6376591

[CR20] Karataş, R. D., Altınöz, A. E., & Eşsizoğlu, A. (2020). Post-traumatic stress disorder and related factors among female victims of sexual assault required to attend a university hospital in Turkey: A cross-sectional cohort study. *Criminal Behaviour and Mental Health,**30*(2–3), 79–94. 10.1002/cbm.2145 32307807 10.1002/cbm.2145

[CR21] Kilpatrick, D. G., Amstadter, A. B., Resnick, H. S., & Ruggiero, K. J. (2007). Rape-related PTSD: Issues and interventions. *Psychiatric Times,**24*(7), 50.

[CR22] Koss, M. P., Bailey, J. A., Yuan, N. P., Herrera, V., & Lichter, E. (2003). Depression and PTSD in survivors of male violence: Research and training initiatives to facilitate recovery. *Psychology of Women Quarterly,**27*(2), 130–142. 10.1111/1471-6402.00093

[CR23] Lindquist, C., Barrick, K., Krebs, C., Crosby, C. M., Lockard, A. J., & Sanders-Phillips, K. (2013). The context and consequences of sexual assault among undergraduate women at historically Black colleges and universities (HBCUs). *Journal of Interpersonal Violence,**28*(12), 2437–2461. 10.1177/0886260513479032 23515164 10.1177/0886260513479032

[CR24] Littleton, H., Grills-Taquechel, A. E., Buck, K., Rosman, L., & Dodd, J. (2013). Health risk behavior and sexual assault among ethnically diverse women. *Psychology of Women Quarterly,**37*(1), 7–21. 10.1177/0361684312451842 24223467 10.1177/0361684312451842 PMC3817570

[CR25] Lôncar, M., Medved, V., Jovanović, N., & Hotujac, L. (2006). Psychological consequences of rape on women in 1991–1995 war in Croatia and Bosnia and Herzegovina. *Croatian Medical Journal,**47*(1), 67–75. 16489699 PMC2080379

[CR26] Luce, H., Schrager, S., & Gilchrist, V. (2010). Sexual assault of women. *American Family Physician,**81*(4), 489–495. 20148503

[CR27] McConnell, A. A., Messman-Moore, T. L., Gratz, K. L., & DiLillo, D. (2020). Beyond the force-substance dichotomy: Examining the experience of combined and incapacitated type rapes and their relation to PTSD symptoms. *Journal of Interpersonal Violence,**35*(23–24), 5853–5876. 10.1177/0886260517724252 29294871 10.1177/0886260517724252

[CR28] Messman-Moore, T., Ward, R. M., Zerubavel, N., Chandley, R. B., & Barton, S. N. (2015). Emotion dysregulation and drinking to cope as predictors and consequences of alcohol-involved sexual assault: Examination of short-term and long-term risk. *Journal of Interpersonal Violence,**30*(4), 601–621. 10.1177/0886260514535259 24919992 10.1177/0886260514535259

[CR29] Mohammed, G. F., & Hashish, R. K. (2015). Sexual violence against females and its impact on their sexual function. *Egyptian Journal of Forensic Sciences,**5*, 96–102. 10.1016/j.ejfs.2014.08.004

[CR30] Nelson, J. D., & Fischer, S. (2021). Recent sexual assault predicting changes in coping motives for alcohol use in first-year college women. *Violence and Victims,**36*(3), 424–435. 10.1891/VV-D-19-00159 34103415 10.1891/VV-D-19-00159

[CR31] Newins, A. R., Glenn, J. J., Wilson, L. C., Wilson, S. M., Kimbrel, N. A., Beckham, J. C., & Calhoun, P. S. (2021). Psychological outcomes following sexual assault: Differences by sexual assault setting. *Psychological Services,**18*(4), 504–511. 10.1037/ser0000426 32271049 10.1037/ser0000426 PMC7544608

[CR32] O’Callaghan, E., Shepp, V., Ullman, S. E., & Kirkner, A. (2019). Navigating sex and sexuality after sexual assault: A qualitative study of survivors and informal support providers. *Journal of Sex Research,**56*(8), 1045–1057. 10.1080/00224499.2018.1506731 30183383 10.1080/00224499.2018.1506731 PMC6401344

[CR33] Page, M. J., McKenzie, J. E., Bossuyt, P. M., Boutron, I., Hoffmann, T. C., Mulrow, C. D., Shamseer, L., Tetzlaff, J. M., Akl, E. A., Brennan, S. E., Chou, R., Glanville, J., Grimshaw, J. M., Hróbjartsson, A., Lalu, M. M., Li, T., Loder, E. W., Mayo-Wilso, E., & Moher, D. (2021). The PRISMA 2020 statement: An updated guideline for reporting systematic reviews. *British Medical Journal,**372*(n71), 1–9. 10.1136/bmj.n71 10.1136/bmj.n71 PMC8005924 33782057

[CR34] Pegram, S. E., & Abbey, A. (2019). Associations between sexual assault severity and psychological and physical health outcomes: Similarities and differences among African American and Caucasian survivors. *Journal of Interpersonal Violence,**34*(19), 4020–4040. 10.1177/0886260516673626 27754921 10.1177/0886260516673626 PMC7019196

[CR35] Perilloux, C., Duntley, J. D., & Buss, D. M. (2012). The costs of rape. *Archives of Sexual Behavior,**41*(5), 1099–1106. 10.1007/s10508-011-9863-9 21975924 10.1007/s10508-011-9863-9

[CR36] Petrak, J., Doyle, A. M., Williams, L., Buchan, L., & Forster, G. (1997). The psychological impact of sexual assault: A study of female attenders of a sexual health psychology service. *Sexual and Marital Therapy,**12*(4), 339–345. 10.1080/02674659708408177

[CR37] Pico-Alfonso, M. A., Echeburúa, E., & Martínez, M. (2008). Personality disorder symptoms in women as a result of chronic intimate male partner violence. *Journal of Family Violence,**23*(7), 577–588. 10.1007/s10896-008-9180-9

[CR38] Pinsky, H. T., Shepard, M. K., Bird, E. K., Gilmore, A. K., Norris, J., Davis, K. C., & George, W. H. (2017). Differences in mental health and sexual outcomes based on type of nonconsensual sexual penetration. *Violence against Women,**23*(9), 1039–1054. 10.1177/1077801216655624 27486127 10.1177/1077801216655624 PMC5225245

[CR39] Pohane, P. U., Jaiswal, S. V., Vahia, V. N., & Sinha, D. (2020). Psychopathology, perceived social support, and coping in survivors of adult sexual assault: A cross-sectional hospital-based study. *Indian Journal Psychiatry,**62*(6), 718–722. 10.4103/psychiatry.IndianJPsychiatry_432_19 10.4103/psychiatry.IndianJPsychiatry_432_19 PMC8052890 33896980

[CR40] Ramos-Lira, L., Saltijeral-Méndez, M. T., Romero-Mendoza, M., Caballero-Gutiérrez, M. A., & Vélez, N. A. M. (2001). Violencia sexual y problemas asociados en una muestra de usuarias de un centro de salud [Sexual violence and associated problems in a sample of female clients of a health center]. *Salud Pública De México,**43*(3), 182–191. 10.1590/s0036-36342001000300002 11452693

[CR41] Rellini, A. H. (2008). Review of the empirical evidence for a theoretical model to understand the sexual problems of women with a history of CSA. *Journal of Sexual Medicine,**5*(1), 31–46. 10.1111/j.1743-6109.2007.00652.x 18069994 10.1111/j.1743-6109.2007.00652.x

[CR42] Rhew, I. C., Stappenbeck, C. A., Bedard-Gilligan, M., Hughes, T. L., & Kaysen, D. (2017). Effects of sexual assault on alcohol use and consequences among young adult sexual minority women. *Journal of Consulting and Clinical Psychology,**85*(5), 424–433. 10.1037/ccp0000202 28287804 10.1037/ccp0000202 PMC5398947

[CR43] Rollison, J., Gore, K., Farris, C., Hero, J., Feistel, K., Akinnirankye, O., Bialas, A., Li, R., & Weilant, S. (2021). *Associations between experiences of sexual assault and/or harassment as an adult and PTSD, depression, and substance use disorders*. University of York.

[CR44] Rothman, K., Salivar, E. G., Roddy, M. K., Hatch, S. G., & Doss, B. D. (2021). Sexual assault among women in college: Immediate and long-term associations with mental health, psychosocial functioning, and romantic relationships. *Journal of Interpersonal Violence,**36*(19–20), 9600–9622. 10.1177/0886260519870158 31423886 10.1177/0886260519870158

[CR45] Stein, M. B., Lang, A. J., Laffaye, C., Satz, L. E., Lenox, R., & Dresselhaus, T. R. (2004). Relationship of sexual assault history to somatic symptoms and health anxiety in women. *General Hospital Psychiatry,**26*(3), 178–183. 10.1016/j.genhosppsych.2003.11.003 15121345 10.1016/j.genhosppsych.2003.11.003

[CR46] Tricco, A. C., Lillie, E., Zarin, W., O’Brien, K. K., Colquhoun, H., Levac, D., Peters, M. D. J., Horsley, T., Weeks, L., Hempel, S., Akl, E. A., Chang, C., McGowan, J., Stewart, L., Hartling, L., Aldcroft, A., Wilson, M. G., Garritty, C., & Straus, A. E. (2018). PRISMA extension for scoping reviews (PRISMAScR): Checklist and explanation. *Annals of Internal Medicine,**169*, 467–473. 10.7326/M18-0850 30178033 10.7326/M18-0850

[CR47] Ullman, S. E. (2004). Sexual assault victimization and suicidal behavior in women: A review of the literature. *Aggression and Violent Behavior,**9*(4), 331–351. 10.1016/s1359-1789(03)00019-3

[CR48] Ullman, S. E., Townsend, S. M., Filipas, H. H., & Starzynski, L. L. (2007). Structural models of the relations of assault severity, social support, avoidance coping, self-blame, and PTSD among sexual assault survivors. *Psychology of Women Quarterly,**31*(1), 23–37. 10.1111/j.1471-6402.2007.00328.x

[CR49] van Berlo, W., & Ensink, B. (2000). Problems with sexuality after sexual assault. *Annual Review of Sex Research,**11*, 235–257. 11351833

[CR50] van der Kolk, B. (2014). *The body keeps the score: Brain, mind, and body in the healing of trauma*. Penguin.

[CR51] Vickerman, K. A., & Margolin, G. (2009). Rape treatment outcome research: Empirical findings and state of the literature. *Clinical Psychology Review,**29*(5), 431–448. 10.1016/j.cpr.2009.04.004 19442425 10.1016/j.cpr.2009.04.004 PMC2773678

[CR52] Vidal, M. E., & Petrak, J. (2007). Shame and adult sexual assault: A study with a group of female survivors recruited from an East London population. *Sexual and Relationship Therapy,**22*(2), 159–171. 10.1080/14681990600784143

[CR53] Vizzotto, A., Oliveira, A. M., Elkis, H., Cordeiro, Q., & Buchain, P. (2013). Psychosocial Characteristics. In M. D. Gellman & J. R. Turner (Eds.), *Encyclopedia of behavioral medicine* (pp. 1578–1580). Springer. 10.1007/978-1-4419-1005-9_918

[CR54] Wadsworth, P., Krahe, E., & Searing, K. (2018). An ecological model of well-being after sexual assault. *Family & Community Health,**41*(1), 37–46. 10.1097/fch.0000000000000168 29135793 10.1097/FCH.0000000000000168

[CR56] World Health Organization. (2021). *Violence against women prevalence estimates, 2018: Global, regional and national prevalence estimates for intimate partner violence against women and global and regional prevalence estimates for non-partner sexual violence against women. Executive summary*. Geneva: World Health Organization.

[CR55] World Health Organization & Pan American Health Organization. (2012). *Understanding and addressing violence against women: Intimate partner violence*. Geneva: World Health Organization.

[CR57] Yeh, M. S. L., Poyares, D., Coimbra, B. M., Mello, A. F., Tufik, S., & Mello, M. F. (2021). Subjective and objective sleep quality in young women with posttraumatic stress disorder following sexual assault: A prospective study. *European Journal of Psychotraumatology,**12*(1), 2–12. 10.1080/20008198.2021.1934788 10.1080/20008198.2021.1934788 PMC8231348 34221253

[CR58] Young, S. L., & Maguire, K. C. (2003). Talking about sexual violence. *Women and Language,**26*(2), 40–52.

